# Predictors of COVID-19 Vaccination Likelihood Among Reproductive-Aged Women in the United States

**DOI:** 10.1177/00333549221081123

**Published:** 2022-03-03

**Authors:** Sirena Gutierrez, Rachel Logan, Cassondra Marshall, Jennifer Kerns, Nadia Diamond-Smith

**Affiliations:** 1 Department of Epidemiology and Biostatistics, University of California, San Francisco, San Francisco, CA, USA; 2 The Equity Experience, LLC, Tampa, FL, USA; 3 Division of Community Health Sciences, School of Public Health, University of California, Berkeley, Berkeley, CA, USA; 4 Department of Obstetrics, Gynecology and Reproductive Sciences, University of California, San Francisco, San Francisco, CA, USA

**Keywords:** COVID-19, coronavirus, vaccine uptake, mistrust

## Abstract

**Objectives:**

Vaccination for COVID-19 is an effective method of preventing complications; however, studies suggest that public attitudes toward the vaccine are heterogeneous. The objective of our study was to identify predictors for low likelihood of COVID-19 vaccination among women in the United States and determine whether reasons for low intention were modified by race, ethnicity, or other characteristics to better understand the factors that shape attitudes toward the COVID-19 vaccine and help inform multilevel interventions.

**Methods:**

In January 2021, we used social media to recruit a cross-section of reproductive-aged women in the United States (N = 5269). Our primary outcome was self-reported low vaccination likelihood (responses of unlikely or very unlikely on a 5-item scale). Our secondary outcome was concerns influencing vaccination decision that participants selected from a list of 19 items. We estimated multivariable logistic regression models and controlled for respondents’ sociodemographic characteristics.

**Results:**

Overall, race and ethnicity, educational attainment, health insurance type, annual household income, partnership status, and US region were associated with low vaccine likelihood. The adjusted odds of reporting low likelihood were 1.83 (95% CI, 1.45-2.32) times greater among non-Hispanic Black than among non-Hispanic White participants. Among pregnant or postpartum participants, breastfeeding status was the strongest predictor (adjusted odds ratio = 2.77; 95% CI, 2.02-3.79).

**Conclusions:**

Vaccine hesitancy and concerns may exacerbate existing COVID-19 health disparities in racial and ethnic groups and highlight the need to target messaging to specific populations, including pregnant and breastfeeding women, because these populations are at high risk for COVID-19 complications.

Vaccination against COVID-19 is the main method to prevent complications from the disease, given the lack of approved pharmaceutical therapeutics as of October 2020.^1^ Concerns about vaccine hesitancy and mistrust were raised as a major global threat by the World Health Organization before the pandemic’s onset and were further intensified by contradicting messaging and false information.^2^ By identifying specific populations and the underlying factors contributing to vaccine hesitancy and mistrust, vaccination strategies and messaging may be improved to change the tide of the pandemic.

As immunization efforts increase, initial reports suggest that COVID-19 vaccination intentions are mixed. In late 2020, US surveys showed that 56% to 69% of adult respondents indicated they would receive the vaccine.^3^ Factors associated with unwillingness to receive the vaccine were being a woman, being of a younger age, being African American or Hispanic, and having less education.^4,5^ A systematic review highlighted that African American and Hispanic populations were more likely than non-Hispanic White populations to experience disproportionately higher rates of SARS-CoV-2 infection and COVID-19–related mortality.^6^ In general, studies indicate that groups that have experienced historical traumas have higher levels of medical mistrust than groups that have not experienced trauma.^7-12^ Specifically, racism, prior interactions with the health care system, and perceived vaccine and disease risk were significant predictors of trust in the COVID-19 vaccine among Black and Latinx adults surveyed in September 2020.^13^ These experiences contextualize and underscore the potential for the pandemic to further exacerbate existing inequalities in socially marginalized groups, including racial and ethnic minority populations, if vaccine concerns and mistrust are not addressed.

Pregnant people are an especially important population for COVID-19 vaccination. COVID-19 increases the risk of preterm birth, stillbirth, and preeclampsia in pregnancy; however, preliminary evidence suggests that the vaccine is safe during pregnancy,^14-16^ and recommendations from the American College of Obstetricians and Gynecologists have supported vaccination during pregnancy since January 2021.^17^ Despite the growing literature and the possibility of severe health outcomes, skepticism about the limited research on safety and effectiveness, concerns about infertility and exposing their developing baby to harmful side effects, and distrust of the health care system may shape vaccine acceptance and confidence among pregnant people. A survey of vaccine acceptance among pregnant women in 16 countries found that women in the United States had some of the lowest levels of vaccine acceptance globally.^18^ Understanding more about barriers to uptake among pregnant people, and how that may intersect with other factors such as race and ethnicity, is key for targeting campaigns and increasing uptake among this population at high risk of complications and adverse pregnancy outcomes from COVID-19.

This study aims to describe COVID-19 vaccination concerns among a cohort of reproductive-aged women and explore the role of sociodemographic factors and pregnancy status on vaccination intention. We hypothesized that women in racial and ethnic minority groups, particularly Black, Hispanic, and multiracial or mixed-race groups, would be less likely than non-Hispanic White women to accept the vaccine and self-report higher levels of various concerns (ie, safety, benefits, lack of information or trust). However, we hypothesized that vaccine intentions and concerns were modified by the participant’s current pregnancy status, given the evolving vaccination guidance for pregnant or postpartum people.

## Methods

### Study Population

A cross-section of English- and Spanish-speaking women (self-identified) aged 18-45 years was recruited in January 2021 via Facebook and Instagram advertisements as part of a larger study, COVID-19’s Impacts on Reproduction in the United States.^19^ Given the tendency for social media recruitment to skew toward a non-Hispanic White population, the recruitment advertisements were designed to oversample non-White women. In addition, because of the parent study’s focus on the impact of COVID-19 on reproductive and maternal health care access, we aimed to overrecruit women living in the South or Midwest, where we hypothesized there would be a greater impact of COVID-19 and related complications. This study was approved by the University of California, San Francisco Institutional Review Board.

Of the 7552 respondents who completed the questionnaire, we excluded those aged <18 or >45 years (n = 130). In addition, we did not include respondents who were sterilized (n = 93) in the primary analytical sample. To maximize the quality of data, we excluded respondents with a survey completion time of <60 seconds (n = 584) and duplicate entries (n = 216).^20^ We also excluded respondents missing data on vaccine hesitancy concerns (n = 1075 [747 of whom were vaccinated]) and any covariate information (n = 169).

### Outcomes

Likelihood of receiving the vaccine, our primary outcome of interest, was assessed in the questionnaire by asking participants, “When a COVID-19 vaccine is available, how likely are you to want to receive the vaccination?” We measured responses by using a 5-item Likert scale (1 = very likely; 2 = likely; 3 = I do not know yet; 4 = unlikely; 5 = very unlikely); we categorized responses of unlikely or very unlikely as low likelihood.

Concerns influencing vaccination decision, our second outcome of interest, were assessed in the questionnaire by asking participants to select all concerns, from a list of 19 responses, that might make them less likely to get the vaccine. Alternatively, they could report that nothing will make them less likely to get the vaccine.

### Covariates

Sociodemographic characteristics included age (18-24, 25-34, 35-45 years), race and ethnicity, educational attainment (≤high school graduate, some college, ≥college degree), health insurance type (public, private, none, other), annual household income (≤$24 999, $25 000-$49 999, $50 000-$74 999, $75 000-$99 999, ≥$100 000), partnership status (cohabiting/married, single, in a relationship but not cohabitating, divorced/separated/other), US region (West, Midwest, Southwest, Southeast, and Northeast, derived from zip codes^21^), and pregnancy status (yes, no/not sure). Respondents could report multiple racial and ethnic categories; however, these respondents were categorized as mixed race. Therefore, we included only those who self-identified as non-Hispanic White, non-Hispanic Black, non-Hispanic Asian, or Hispanic in those categories. Because of small sample sizes, we used “other” racial category for respondents who selected American Indian/Alaska Native and Native Hawaiian/Other Pacific Islander.

### Statistical Analysis

We generated descriptive statistics to describe the demographic characteristics of participants. To test differences in concerns influencing vaccination decision among racial and ethnic groups, we conducted Pearson χ^2^ tests of independence. Multivariable logistic regression models were adjusted for age, race and ethnicity, educational attainment, health insurance type, annual household income, partnership status, US region, and pregnancy status. We also restricted the models to women who were currently pregnant or postpartum during the questionnaire, because we hypothesized that their vaccination intentions and concerns would differ from those of the overall sample. We conducted a sensitivity analysis by including respondents who were not sure of their vaccine likelihood in the unlikely/very unlikely category. All *P* values were 2-sided; α = .05 was the cutoff for significance. We performed all statistical analyses using Stata version 16 (StataCorp LLC).

## Results

Of the 5269 respondents who participated in the questionnaire, most were non-Hispanic White (58.8%), aged 25-34 years (53.3%), had ≥college degree (52.3%), had private health insurance (58.5%), were either cohabiting or married (75.5%), and were not currently pregnant (85.5%) (Table 1). Sociodemographic patterns among pregnant and postpartum participants (n = 1190) were similar to those of the overall group; however, they were significantly different in some ways. They were younger (80.1% vs 68.6% aged <35 years) and more likely to be cohabiting or married (91.6% vs 75.5%). Overall, the distribution of responses for COVID-19 vaccine likelihood was very likely (40.3%), somewhat likely (16.8%), not sure (22.1%), unlikely (7.6%), and very unlikely (13.3%) (Table 1).

**Table 1.** Baseline characteristics of participants recruited for the COVID-19’s Impacts on Reproduction in the United States study, January 2021^a^

Characteristic	Overall, no. (%) (N = 5269)	Pregnant or postpartum, no. (%) (n = 1190)
Race and ethnicity		
Non-Hispanic White	3099 (58.8)	785 (66.0)
Non-Hispanic Black	386 (7.3)	94 (7.9)
Non-Hispanic Asian	527 (10.0)	61 (5.1)
Hispanic	764 (14.5)	148 (12.4)
Mixed race	418 (7.9)	77 (6.5)
Other^b^	75 (1.4)	25 (2.1)
Age, y		
18-24	807 (15.3)	124 (10.4)
25-34	2807 (53.3)	829 (69.7)
35-45	1655 (31.4)	237 (19.9)
Educational attainment		
≤High school graduate	970 (18.4)	236 (19.8)
Some college	1544 (29.3)	342 (28.7)
≥College degree	2755 (52.3)	612 (51.4)
Health insurance type		
Private	3080 (58.5)	704 (59.2)
Public	1580 (30.0)	411 (34.5)
None	554 (10.5)	71 (6.0)
Other	55 (1.0)	4 (0.3)
Annual household income, $		
≥100 000	1078 (20.5)	293 (24.6)
75 000-99 999	721 (13.7)	180 (15.1)
50 000-74 999	1109 (21.1)	264 (22.2)
25 000-49 999	1221 (23.2)	276 (23.2)
≤24 999	1140 (21.6)	177 (14.9)
Partnership status		
Cohabiting or married	3977 (75.5)	1090 (91.6)
Single	634 (12.0)	39 (3.3)
In a relationship but not cohabiting	541 (10.3)	47 (4.0)
Divorced/separated/other	117 (2.2)	14 (1.2)
US region		
West	1167 (22.2)	301 (25.3)
Midwest	1068 (20.3)	229 (19.2)
Southwest	644 (12.2)	163 (13.7)
Southeast	1629 (30.9)	335 (28.2)
Northeast	761 (14.4)	162 (13.6)
Currently pregnant		
No or not sure	4505 (85.5)	426 (35.8)
Yes	764 (14.5)	764 (64.2)
Likelihood of wanting the COVID-19 vaccine		
Very likely	2124 (40.3)	363 (30.5)
Likely	884 (16.8)	272 (22.9)
I do not know yet	1165 (22.1)	293 (24.6)
Unlikely	398 (7.6)	125 (10.5)
Very unlikely	698 (13.3)	137 (11.5)

a Data obtained from participants who completed surveys that included vaccine-related questions in January 2021.^19^

b Includes self-reported “other” category, American Indian/Alaska Native, and Native Hawaiian/Other Pacific Islander.

After full adjustment for sociodemographic factors, the variables associated with low vaccine likelihood were race and ethnicity, educational attainment, health insurance type, annual household income, partnership status, and US region. Compared with non-Hispanic White participants, non-Hispanic Black participants had significantly higher odds of reporting low vaccination likelihood (adjusted odds ratio [aOR] = 1.83; 95% CI, 1.45-2.32) (Table 2). Non-Hispanic Asian and Hispanic race and ethnicity were associated with lower odds of low vaccination likelihood (aOR = 0.38 [95% CI, 0.27-0.55] and aOR = 0.66 [95% CI, 0.53-0.83], respectively). Lower levels of education (≤high school degree or some college, compared with ≥college degree) was associated with higher odds of low vaccination likelihood (aOR = 1.59 [95% CI, 1.29-1.95] and aOR = 1.59 [95% CI, 1.33-1.88], respectively). Public health insurance was associated with higher odds of low vaccination likelihood (aOR = 1.42; 95% CI, 1.18-1.70) compared with private health insurance. Lower income was associated with higher odds of vaccine hesitancy compared with an annual household income of ≥$100 000, with the highest odds among those in the lowest income category of <$24 999 (aOR = 2.27; 95% CI, 1.70-3.02). Living in the Midwest or Southeast was associated with higher odds of low vaccination likelihood (aOR = 1.53 [95% CI, 1.22-1.92] and aOR = 1.68 [95% CI, 1.36-2.06], respectively), compared with those residing in the West. Being single, in a relationship but not living with a partner, and divorced/separated/other were associated with lower odds of low vaccination likelihood, compared with those who were cohabiting or married. In the sensitivity analysis that included respondents who were not sure of their vaccine intention, predictors of low vaccine likelihood followed similar patterns; however, women who were currently pregnant were less likely than women who were not pregnant to want to receive the vaccine (aOR = 1.19; 95% CI, 1.01-1.41).

**Table 2.** Multivariate model of predictors of low likelihood of wanting to receive the COVID-19 vaccine, among participants overall and pregnant or postpartum participants in the COVID-19’s Impacts on Reproduction in the United States study, January 2021^a^

Characteristic	Overall (N = 5269)		Pregnant or postpartum (n = 1190)	
	Adjusted odds ratio^b^ (95% CI)	*P* value^c^	Adjusted odds ratio^b^ (95% CI)	*P* value^c^
Race and ethnicity				
Non-Hispanic White	1 [Reference]	—	1 [Reference]	—
Non-Hispanic Black	1.83 (1.45-2.32)	<.001	1.51 (0.90-2.54)	.12
Non-Hispanic Asian	0.38 (0.27-0.55)	<.001	1.06 (0.49-2.28)	.88
Hispanic	0.66 (0.53-0.83)	<.001	0.65 (0.38-1.12)	.12
Mixed race	1.05 (0.81-1.35)	.73	1.29 (0.73-2.29)	.38
Other^d^	1.12 (0.65-1.93)	.68	0.68 (0.19-2.40)	.55
Age, y				
18-24	0.98 (0.78-1.24)	.89	2.37 (1.34-4.17)	.003
25-34	0.98 (0.84-1.15)	.85	1.53 (1.00-2.32)	.05
35-45	1 [Reference]	—	1 [Reference]	—
Educational attainment				
≤High school graduate	1.59 (1.29-1.95)	<.001	1.23 (0.77-1.97)	.38
Some college	1.59 (1.33-1.88)	<.001	1.64 (1.13-2.38)	.009
≥College degree	1 [Reference]	—	1 [Reference]	—
Health insurance type				
Private	1 [Reference]	—	1 [Reference]	—
Public	1.42 (1.18-1.70)	<.001	0.78 (0.53-1.14)	.20
None/other	1.14 (0.89-1.45)	.30	0.43 (0.19-0.97)	.04
Annual household income, $				
≥100 000	1 [Reference]	—	1 [Reference]	—
75 000-99 999	1.67 (1.27-2.21)	<.001	1.68 (0.97-2.90)	.06
50 000-74 999	1.80 (1.39-2.32)	<.001	1.91 (1.16-3.15)	.01
25 000-49 999	1.79 (1.38-2.34)	<.001	2.40 (1.42-4.06)	.001
≤24 999	2.27 (1.70-3.02)	<.001	4.74 (2.54-8.86)	<.001
Partnership status				
Cohabiting or married	1 [Reference]	—	1 [Reference]	—
Single	0.71 (0.56-0.90)	.004	1.27 (0.57-2.80)	.56
In a relationship, but not living with partner	0.75 (0.59-0.96)	.03	0.65 (0.30-1.40)	.27
Divorced/separated/other	0.61 (0.37-0.99)	.05	0.46 (0.09-2.26)	.34
US region				
West	1 [Reference]	—	1 [Reference]	—
Midwest	1.53 (1.22-1.92)	<.001	1.90 (1.18-3.05)	.008
Southwest	1.23 (0.95-1.61)	.12	1.12 (0.65-1.94)	.68
Southeast	1.68 (1.36-2.06)	<.001	2.02 (1.31-3.12)	.002
Northeast	0.95 (0.73-1.24)	.72	1.13 (0.64-2.00)	.67
Is currently pregnant				
No	1 [Reference]	—	—	—
Yes	1.02 (0.84-1.25)	.83	—	—
Is currently or will be breastfeeding				
No	—	—	1 [Reference]	—
Yes	—	—	2.77 (2.02-3.79)	<.001

Abbreviation: —, not applicable.

a Data obtained by participants who completed the surveys that contained vaccine-related questions in January 2021.^19^

b Reference category were those who stated very likely, likely, and I do not know yet for their likelihood of receiving the COVID-19 vaccine.

c All *P* values were 2-sided; α = .05 was the cutoff for significance.

d Includes self-reported “other” category, American Indian/Alaska Native, and Native Hawaiian/Other Pacific Islander.

Among women who were currently pregnant or postpartum, the variables associated with low vaccination likelihood were age, educational attainment, health insurance type, annual household income, US region, and breastfeeding status. We found higher odds of low vaccination likelihood among pregnant or postpartum women aged 18-24 years than among women aged 35-45 years (aOR = 2.37; 95% CI, 1.34-4.17) and women currently breastfeeding or planning to breastfeed (aOR = 2.77; 95% CI, 2.02-3.79) compared with women who were not. In addition, similar to results for overall participants, we found higher odds (compared with their reference groups) of low vaccination likelihood among pregnant or postpartum women with lower levels of education and income and those living in the Midwest or Southeast (Table 2).

Overall, the distribution of reporting no vaccine concerns by racial and ethnic group was the following: non-Hispanic Asian (40.6%), non-Hispanic White (30.6%), Hispanic (27.0%), other (24.0%), mixed race (22.3%), and non-Hispanic Black (11.1%) ( $\chi_{5}^{2}$ = 109.9; *P* < .001). Although we found significant differences among racial and ethnic groups in items influencing vaccination likelihood, the top 3 concerns were the same: “I do not trust the vaccine,” “It depends on the risks/adverse events,” and “I need more information first,” despite a greater percentage of non-Hispanic Black participants reporting vaccine-related concerns than participants in other racial and ethnic groups. In addition, a greater percentage of non-Hispanic Black participants indicated that they did not trust the vaccine (45.9%) compared with participants in other racial and ethnic groups. Compared with non-Hispanic White participants, non-Hispanic Black participants reported nearly twice the percentage of vaccine mistrust (25.6%) and non-Hispanic Asian participants reported almost 4 times the percentage of vaccine mistrust (12.0%) (Table 3).

**Table 3.** Concerns influencing vaccination decision among reproductive-aged women participating in the COVID-19’s Impacts on Reproduction in the United States study (N = 5269), by race and ethnicity, January 2021^a^

Concern	Race and ethnicity, no. (%)						χ^2^ Test of independence^c^
	Non-Hispanic Black (n = 386)	Non-Hispanic Asian (n = 527)	Hispanic (n = 764)	Non-Hispanic White (n = 3099)	Mixed race (n = 418)	Other^b^ (n = 75)	
Nothing will make me less likely; I will get it as soon as I can	43 (11.1)	214 (40.6)	206 (27.0)	947 (30.6)	93 (22.3)	18 (24.0)	<.001
I will not get/am never sick	31 (8.0)	33 (6.3)	30 (3.9)	104 (3.4)	20 (4.8)	4 (5.3)	<.001
It is just a virus/not fatal/not necessary	28 (7.3)	16 (3.0)	27 (3.5)	174 (5.6)	17 (4.1)	6 (8.0)	.006
I never get vaccinated	54 (14.0)	19 (3.6)	40 (5.2)	178 (5.7)	28 (6.7)	10 (13.3)	<.001
							
I do not trust the vaccine	177 (45.9)	63 (12.0)	197 (25.8)	792 (25.6)	124 (29.7)	11 (14.7)	<.001
I do not want to pay for it	48 (12.4)	50 (9.5)	85 (11.1)	281 (9.1)	41 (9.8)	5 (6.7)	.20
My region is not a high-risk area	20 (5.2)	3 (0.6)	11 (1.4)	100 (3.2)	12 (2.9)	2 (2.7)	<.001
Vaccination location is not convenient	19 (4.9)	27 (5.1)	17 (2.2)	126 (4.1)	17 (4.1)	6 (8.0)	.04
It depends on the risks/adverse events	127 (32.9)	134 (25.4)	210 (27.5)	884 (28.5)	143 (34.2)	13 (17.3)	.003
Vaccination is worse than being ill	25 (6.5)	13 (2.5)	20 (2.6)	132 (4.3)	19 (4.6)	6 (8.0)	.005
I have not thought about it yet	16 (4.2)	20 (3.8)	59 (7.7)	77 (2.5)	11 (2.6)	2 (2.7)	<.001
I am not in a risk group with underlying conditions	21 (5.4)	37 (7.0)	37 (4.8)	276 (8.9)	38 (9.1)	4 (5.3)	.002
I need more information first	114 (29.5)	110 (20.9)	205 (26.8)	721 (23.3)	133 (31.8)	13 (17.3)	<.001
It will not help	15 (3.9)	7 (1.3)	11 (1.4)	70 (2.3)	11 (2.6)	3 (4.0)	.06
							
I have already had COVID-19	16 (4.2)	11 (2.1)	34 (4.5)	114 (3.7)	15 (3.6)	6 (8.0)	.10
I am going to let others get it first (herd immunity)	67 (17.4)	54 (10.3)	87 (11.4)	246 (7.9)	54 (12.9)	6 (8.0)	<.001
I am/will be breastfeeding	46 (11.9)	33 (6.3)	84 (11.0)	608 (19.6)	77 (18.4)	6 (8.0)	<.001
I am pregnant/plan to get pregnant	29 (7.5)	28 (5.3)	58 (7.6)	399 (12.9)	58 (13.9)	6 (8.0)	<.001
Do not know yet	19 (4.9)	18 (3.4)	45 (5.9)	108 (3.5)	22 (5.3)	5 (6.7)	.02
Other	4 (1.0)	10 (1.9)	11 (1.4)	75 (2.4)	14 (3.4)	4 (5.3)	.05

a Data obtained by participants who completed the surveys that contained vaccine-related questions in January 2021.^19^

b Includes self-reported “other” category, American Indian/Alaska Native, and Native Hawaiian/Other Pacific Islander.

c All *P* values were 2-sided; α = .05 was the cutoff for significance.

## Discussion

This study used data from a sample of women of reproductive age surveyed in January 2021 to identify the predictors of vaccine likelihood and to evaluate the main sources of vaccine-related concerns. Women who were less likely (compared with their reference groups) to intend to get the vaccine were non-Hispanic Black, had less education and annual household income, used public health insurance, or lived in the Midwest or Southeast. However, women who were single, divorced, or separated were less likely to have low vaccine likelihood than women who were married or cohabiting with a partner. Among participants who were pregnant or postpartum, the factors associated with low vaccine likelihood were being younger, having some college education, having lower income levels, living in the Midwest or Southeast, or currently breastfeeding or planning to breastfeed. Based on the magnitude of the associations, race and ethnicity appear to be the strongest predictors of low vaccine likelihood in the overall sample, although among pregnant and postpartum participants, current breastfeeding status or intention was the strongest predictor. Across all racial and ethnic groups, participants reported the same top concerns, although greater percentages of non-Hispanic Black, Hispanic, and mixed-race participants had greater levels of concerns.

In our sample, 57.1% of participants stated they were very likely or likely to receive the vaccine, which is comparable to a range of 56.0% to 68.6% reported in US-based studies.^3,22^ Our findings of low vaccine likelihood being associated with race and ethnicity and low socioeconomic status are similar to findings of studies conducted earlier in the pandemic (April and July 2020); however, they noted tailored campaigns with health experts and trusted physicians as opposed to politicized endorsements as a potential mechanism to increase uptake and accessibility of understanding benefits.^4,5^ Although general vaccine mistrust was a top concern across all racial and ethnic groups, it was more prevalent among non-Hispanic Black participants. Despite the consistently elevated levels of vaccine mistrust across all racial and ethnic groups in our study, participants noted the need for more information as a top concern—suggesting that messaging strategies adapted to various racial and ethnic groups and health literacy levels can help mitigate vaccine concerns. Targeted strategies that have been used in the past, such as additional funding for urban-area health departments, partnerships with racial and ethnic minority health organizations, increased Medicaid reimbursements, and the development of local action plans, in conjunction with universal campaigns, were successful in closing the gap in measles vaccine coverage among racial and ethnic minority groups.^23^ Lessons from seasonal influenza immunization efforts to close the racial gap point to a nuanced, multidimensional approach that considers demographic, racial, and ideological differences when targeting messaging to increase vaccine confidence and convenience while combating complacency.^24-26^

Racial and ethnic gaps in immunization place racial and ethnic minority populations at great risk for COVID-19 and related complications, which merits the identification of the drivers of mistrust in a framework that ensures equitable vaccine allocation. While we cannot disentangle the complex reasons of mistrust reported by the study participants, our findings have been consistent with the findings of others who reported that socially marginalized populations, particularly Black and Hispanic populations, have higher odds of vaccine mistrust than non-Hispanic White populations. These COVID-19 and influenza studies indicated that vaccine mistrust was associated with past experiences with the medical system, generalized trust, perceived discrimination, and sociodemographic characteristics.^10-13,25-27^ Despite increased vaccine accessibility as a result of US Food and Drug Administration authorizations, miscommunication at the inception of the pandemic in the context of historical and ongoing injustices contributed to widespread and disproportionate mistrust of the health care system across racial and ethnic groups.^13,28^ These findings suggest that different pathways exist for interventions among racial and ethnic groups, for example, using different types of messaging approaches, engaging different types of spokespeople, and potentially addressing other larger factors that contribute to lack of trust.

The reported geographic differences of lower vaccine likelihood in the Midwest and Southeast are consistent with data collected from the American Community Survey Public Use Microdata Area.^29^ A recent study using US Census data demonstrated that state-level variation in persistent vaccine hesitancy in Mountain States and the South was significantly associated with White but not Black participants, noting that state-level political affinity could be a mechanism for these differences.^30^ Interestingly, our findings were significant even after adjusting for race and ethnicity; furthermore, we suspect that these geographic differences may be rooted in the sociopolitical environment. Although these regional variations in vaccine intentions follow similar patterns of state-level vaccine uptake,^31^ we hypothesize that geography is a correlate of contextual historical, sociocultural, environmental, and political factors that influence personal perceptions of the COVID-19 vaccine. As such, we recognize that future work should differentiate these factors from state-level factors related to access and availability of the vaccine to ameliorate region-specific vaccination concerns.

Among pregnant and postpartum women, planning to or currently breastfeeding was the leading predictor of having lower vaccine intentions. This finding is not surprising because the vaccine development and regulatory approval process progressed rapidly, and the guidelines recommending that pregnant and lactating populations have access to the vaccine were not released by the Centers for Disease Control and Prevention (CDC) until late April 2021.^17^ In September 2021, CDC released a report stating that urgent action was needed to increase vaccination among women who were pregnant, lactating, and considering becoming pregnant, because the benefits of vaccination far outweigh the potential risks.^32^ As of May 2021, 16.3% of pregnant women had received ≥1 dose of a COVID-19 vaccine, with the lowest rates among Hispanic and non-Hispanic Black women.^33^ Disparities in vaccine coverage are not unique to COVID-19, as hesitancy in vaccination uptake for seasonal influenza has been seen among pregnant populations.^34^ Future studies should address the role of health care provider referrals and provide tailored educational messaging during prenatal care to reduce misconceptions among pregnant and postpartum women.

### Limitations

This study had several limitations. First, the use of social media platforms for sample recruitment hindered generalizability to the broader US population. However, these platforms can leverage targeting users through different advertising messaging to garner a heterogeneous population.^35^ Second, we were unable to assess whether significant differences existed between those who viewed the social media advertisements and those who engaged and decided to complete the survey. In prior studies, samples recruited via social media were shown to be fairly representative of their target population, and some researchers suggest that social media recruitment is better than recruitment by other traditional methods in reaching young and hard-to-reach populations.^36^ Our data were collected in January 2021, and we expect that vaccine sentiments may have changed with evolving public health messaging. However, recent data from the Household Pulse Survey indicate that the proportion of respondents who are “strongly hesitant” has not changed over time.^29^ Use of social media recruitment allowed us to collect data quickly at reduced costs during a pandemic when other alternatives were restricted. Lastly, while we controlled for all conceivable factors associated with vaccine uptake that were captured in the questionnaire, other factors, such as the perceived risk and knowledge of COVID-19 and its vaccine, were not included. Also, we did not assess structural factors related to the historical, lifetime, and daily experience of racism and discrimination or cultural nuances within racial and ethnic groups. Despite limited broader generalizations, we were still able to draw inferences about a diverse sample of women using social media in the United States. Fundamentally, we as a country and globe are not going to be able to move through and, hopefully, past, the COVID-19 pandemic without addressing vaccine hesitancy. Understanding the complexities, especially among populations at high risk for COVID-19 complications, such as pregnant and lactating women, and among groups that are socially marginalized, can help shape advocacy campaigns and increase uptake.

## Conclusion

COVID-19 has further exacerbated health inequities related to COVID-19 outcomes for groups socially disadvantaged due to their racial and ethnic identities.^6^ Moving forward, vaccine messaging should focus on honoring and addressing people’s concerns and removing structural barriers to vaccine-related information and services to ensure equitable access to the vaccine. Communication efforts tailored toward groups at high risk of COVID-19 complications, including racial and ethnic minority populations and pregnant and lactating women, must focus on increasing vaccine knowledge and confidence. Moreover, policies and community engagement interventions must work to repair the trust of socially marginalized communities to further improve COVID-19–related health outcomes and reduce disparities.
